# Interaction Mechanisms of Insensitive Explosive FOX-7 and Graphene Oxides from Ab Initio Calculations

**DOI:** 10.3390/nano9091290

**Published:** 2019-09-09

**Authors:** Yan Su, Yuanze Sun, Jijun Zhao

**Affiliations:** Key Laboratory of Materials Modification by Laser, Electron, and Ion Beams (Dalian University of Technology), Ministry of Education, Dalian 116024, China (Y.S.) (Y.S.)

**Keywords:** energetic material–graphene oxide composite, hydrogen bonds, binding energy, band alignment

## Abstract

Energetic material–graphene oxide (EM–GO) composites exhibit excellent thermal stability and insensitivity to mechanical stimuli. The interfacial interactions play an important role in affecting the structural and electrical properties of EM–GO composites. FOX-7 crystal with a wave-shaped layer structure is an ideal prototype system for matching with oxygen-rich GO monolayers to form FOX-7–GO composites. Here, we conducted a systematic investigation on FOX-7–GO composites by dispersion-corrected density functional approach. Our results revealed that there exists relatively strong interaction in the FOX-7–GO interface, which stems from the synergistic effect of interfacial charge transfer and hydrogen bonds. The electronic structure analyses demonstrated that GO can hybridize with FOX-7 to reduce charge accumulation on the FOX-7 surface. These theoretical results are useful for clarifying the interfacial effects on the sensitivity of FOX-7–GO composites.

## 1. Introduction

Energetic materials (EMs) have attracted great research attention for several decades due to their broad range of both military and civilian applications [1,2]. Sensitivity and thermal stability are two important issues of EMs in practical applications. Thus, exploration of new EMs with low sensitivity and high thermal stability is very important and meaningful to improve their performance, as well as their safety in transport and storage. The sensitivity of EMs involves many factors, including molecular and crystalline structure, preliminary temperature, and external stimulus [3,4,5,6]. Among them, molecular and crystal structures, including chemical composition, crystal packing, crystal morphology, size, shape, purity, and defects, are strongly related to the intrinsic qualities of the materials. Until now, various strategies, such as recrystallization, energetic co-crystals, adding and coating of the inert materials including with organic wax [7], high polymers [8], stearic acid [9], and carbon materials (CMs) [10,11,12,13,14,15,16,17,18,19,20,21,22], have been proposed to reduce the sensitivity and improve the performance of EMs.

CMs have garnered significant interest as a candidate coating layer for EMs due to their intriguing structures and unique properties. Jin et al. found that by adding 1 wt. % fullerene to HMX (1,3,5,7-tetranitro-1,3,5,7-tetrazocine), the friction and impact sensitivities of HMX were reduced from 100% to 70% and to 60%, respectively [10]. Furthermore, by introducing 1.0 wt. % graphene oxide (GO) and 1.0 wt. % graphite, both the sensitivity and the friction of HMX were reduced from 100% to 8% and to 0%, respectively [11]. Li et al. and Liu et al. separately confirmed that GO sheets not only notably reduced the sensitivity, but also enhanced the thermal stability of HMX [12,13]. By solvent–anti-solvent method, Wang et al. demonstrated that GO sheets were well coated on the surface of TKX-50 crystal (dihydroxylammonium 5,5′-bistetrazole-1,1′-diolate, a new azole-based high-performance and low-sensitivity high explosive crystal) and formed TKX-50–GO composite with reduced sensitivity, but with no obvious effect on the thermal stability [14].

Impregnation or conjugation of other energetic complexes with GO is also widely regarded as a successful strategy for improving thermal stability and insensitivity. After being doped with 0.5% GO, the laser ignition and burning rates of nitrocellulose (NC) films were obviously improved [15]. Meanwhile, the higher activation energies of decomposition also indicated a higher thermal stability. Recently, Jiang et al. further demonstrated that GO was an effective additive to enhance the energetic performance of aluminum with a high specific energy density [16]. Based on GO, Pang and Gozin coordinated nitrogen-rich energetic ligands and metal ions to form new energetic coordination nanomaterials, which had high thermal stability and insensitivity to mechanical stimuli [17,18].

The atomistic mechanism behind GO’s improvement of the thermal stability and insensitivity of EMs, including explosives, propellants, and pyrotechnics, is interesting. It has been shown that through hydrogen bonding interactions, the abundant oxygenic functional groups on the basal sheet and edges of GO can readily mediate adhesion and shear interactions of EM–GO interfaces. Hydrogen bonding can effectively dissipate mechanical shock and heat at the molecular level, thus preventing the formation of hot spots. First-principles calculation suggested that in carbon nanostructures and graphene layers, the energetic molecules of FOX-7 (1,1-Diamino-2,2-dinitroethylene), RDX (hexahydro-1,3,5-trinitro-striazine), and HMX can be stabilized, thereby reducing the sensitivity [19]. Recent molecular dynamic simulations and kinetics calculations using ReaxFF have also indicated that as an efficient desensitizer or additive, GO could increase the molecular stability and decomposition activation energy of ATRZ (4,4′-Azo-1,2,4-triazole, a new stable high-nitrogen compound) [20]. Ab initio molecular dynamics simulations by Liu et al. showed that functionalized graphene with a carbon vacancy defect could greatly accelerate the thermal decomposition of nitromethane and its derivatives [21]. Moreover, using nonequilibrium reactive molecular dynamics, Lee et al. studied the explosion dynamics of nitromethane confined in a carbon nanotube nanocontainer, which provided some new insights into nanoscale explosions [22].

All these previous studies have provided important information concerning the thermal stability, desensitizing effect, and thermal decomposition mechanism of EM–CM, especially EM–GO composites via coating or encapsulation methods. However, understanding of how the GO affects the stability and sensitivity of coated EMs at the atomic level has not yet been elucidated, due to its immense complexity. Thus, we were motivated to investigate the interfacial interactions and electronic structure between EMs and GO. FOX-7, a highly explosive compound with excellent performance and low initiation sensitivity, has been widely used in munitions and low vulnerability ammunition compositions [23]. FOX-7 crystal has a wave-shaped layer structure, in which hydrogen bonds and van der Waals interactions dominate the interlayer interactions. Therefore, it is an ideal prototype system for matching with oxygen-rich GO monolayers to form FOX-7–GO composites. In this paper, we present a systematic exploration of the interfacial interaction between a FOX-7 monolayer and GO monolayer with different oxygenic functional groups, as well as the structural and electrical properties thereof. Our results might can offer references with which to assess the desensitizing effect of GO on FOX-7 and other energetic material particle surfaces.

## 2. Computational Methods and Models

All first-principles calculations were carried out using the Vienna Ab Initio Simulation Package (VASP) based on spin-polarized density functional theory (DFT) [24]. We chose the Perdew–Burke–Ernzerhof (PBE) functional within the generalized gradient approximation (GGA) to treat the exchange–correlation interaction [25]. The electron-ion interactions were described by the projector augmented wave (PAW) potentials [26]. To take into account the long-range van der Waals (vdW) interactions between GO and FOX-7 layers, a semi-empirical dispersion-corrected DFT-D3 scheme proposed by Grimme was adopted [27]. The energy cutoff for plane-wave basis was set at 500 eV. To sample the Brillouin zone (BZ) of reciprocal space, we used the Γ-centered *k* point grids of 3 × 3 × 1 for structural optimizations and 9 × 9 × 1 for band structures. The present calculations were employed with the static density functional theory at 0 K, without considering the entropy role at different temperatures.

**Figure 1:**
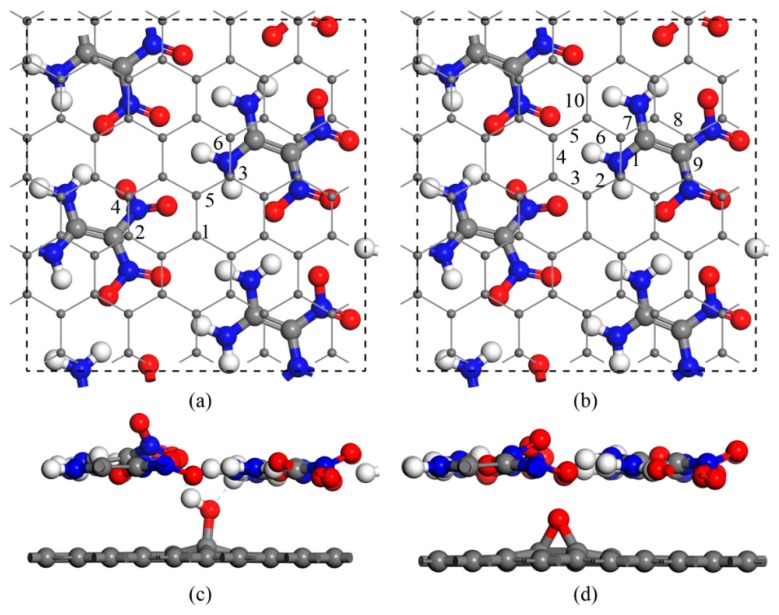
Schematic of the 1,1-Diamino-2,2-dinitroethylene–graphene oxide (FOX-7–GO) composites with different oxygen-containing groups on GO. (**a**) Top view for the six types of single hydroxyl group on GO. (**b**) Top view for the 10 types of single epoxy group on GO. (**c,d**) Side views of the two stable configurations. Gray, red, and white balls represent carbon, oxygen, and hydrogen atoms, respectively.

The GO models were constructed by randomly adding epoxy and hydroxyl groups on the graphene basal plane according to the widely accepted Lerf–Klinowski rules [28]. The O:C ratio on the graphene plane was controlled at 1.67% and 43.33%. We selected seven energetically favorable GO models with different hydroxyl/epoxy ratios, as suggested in our previous work [29]. The constructed GO models consisting of 60 carbon atoms had a rectangular shape with a size of 1.27 × 1.32 nm. Meanwhile, the lattice parameters of FOX-7 monolayer were 0.694 nm, 0.657 nm, and 1.131 nm from the present calculation, which was consistent with the experimental values [30]. As shown in Figure 1, the hybrid FOX-7–GO system was constructed by stacking a GO layer and a 2 × 2 FOX-7 layer. The lattice parameters of the hybrid system were adopted the GO values, since the lattice mismatch was only 4% and 5% in the *x* and *y* directions, respectively. Note that as a compromise between computational cost and accuracy, this computational scheme has been proven to be reliable in handling composite systems of energetic materials [19,20,21].

## 3. Results and Discussion

Based on the optimized structures of FOX-7–GO composites, we quantitatively investigated the interfacial properties between GO and FOX-7. The interaction strength of FOX-7–GO can be characterized by the binding energy defined as

(1) $$\Delta E_{b}=\left(E_{FOX-7–GO\;}-E_{GO}-E_{FOX-7}\right)/S$$

where $E_{FOX-7–GO}$ is the total energy of FOX-7–GO composite; $E_{GO}$, $E_{FOX-7}$ are the total energies of isolated GO monolayer and isolated FOX-7 monolayer, respectively; and *S* is the interface area in the plane. By definition, a negative value of $\Delta E_{b}$ means that the GO and FOX-7 monolayers can combine spontaneously, and a lower value represents a more stable composite configuration.

We first considered multiple interaction configurations of GO with a single hydroxyl (G–OH) or an epoxy group (G–O) to identify the stability of the composites. According to the interfacial interaction sites with respect to the amino or nitro group on FOX-7, 6 and 10 different configurations were constructed for G–OH and G–O, respectively (see Figure 1 for the top and side views). In the composites, the interfacial interaction distorted the amino or nitro group and thus destroyed the wave-shaped layer of FOX-7. The hydrogen bonds and binding energies between the GO and FOX-7 monolayers for all composites are summarized in Table 1. For G–OH, the binding energies with the FOX-7 monolayer ranged from −0.49 to −0.70 eV·nm^−2^. For G–O, however, the binding energies increased to over −1.11 eV·nm^−2^, which was close to that of FOX-7–graphene (FOX-7–G) and indicated a relatively stronger interaction in FOX-7–G–O, but no hydrogen bonds were formed. This difference in binding energy was probably due to the fact that the hydroxyl group in FOX-7–G–OH made the interface equilibrium distances larger than in FOX-7–G–O or FOX-7–G.

**Figure 2:**
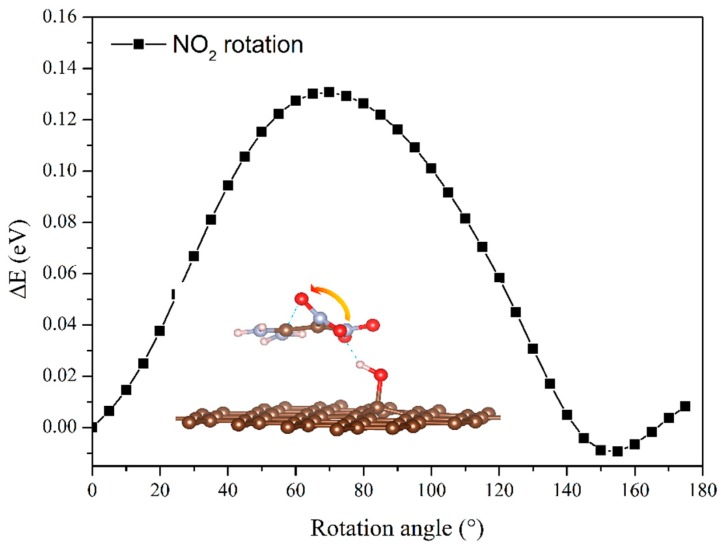
Evolution of hydrogen bond strength between FOX-7 and the –OH group of GO with the rotation angle of the –NO_2_ group.

**Figure 3:**
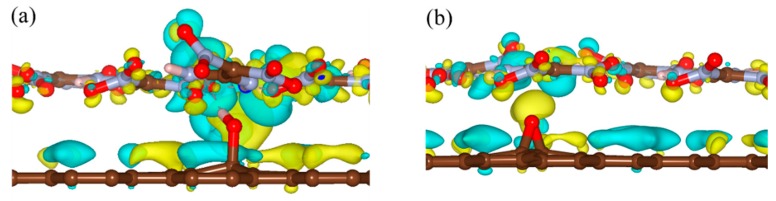
Charge density differences of (**a**) FOX-7–G–OH and (**b**) FOX-7–G–O composites. Isosurface levels were set at 0.0015 Bohr^−3^; cyan: charge accumulation; yellow: charge depletion.

To clarify the effects of the hydrogen bond on the interaction energy of EM–GOs, we took the FOX-7–G–OH (site1) as an example and investigated the hydrogen bond strength between FOX-7 and the –OH group of GO. As shown in the inset of Figure 2, with the rotation of –NO_2_ group, the hydrogen bond broke from the first O atom and formed with the other O atom. Here, we have defined the bond strength as: ∆*E*^φ^ = *E^φ^*_FOX-7–G–OH_ - *E*^φ^_FOX-7_, in which *φ* is the rotation angle, and *E*^φ^_FOX-7–G–OH_ and *E*^φ^_FOX-7_ are the energies of the FOX-7–G–OH (site1) hybrid system and the FOX-7 monolayer at a particular angle *φ*, respectively. Figure 2 plots ∆*E*^φ^ with *φ* ranging from 0° to 180°, in which ∆*E*^φ^ has been rescaled by setting ∆*E*^0^ = 0. It is noteworthy that because of the interaction between the nitro group and the other groups inside the FOX-7 molecule, the periodicity here is not 180°, but about 150°. With the increase of *φ*, ∆*E*^φ^ increased to 0.13 eV and decreased to −0.03 eV again, corresponding to the breaking and re-forming of the hydrogen bond. Clearly, the form of the hydrogen bond can increase the binding energy of the FOX-7–GO composite. The charge density differences can help further clarify the interfacial interactions between FOX-7 and GO with a single hydroxyl or epoxy group. For the most stable configurations of FOX-7–G–OH and FOX-7–G–O composites (site1 for G–OH and site 5 for G–O, see Figure 1 and Table 1), the charge density differences can be found in Figure 3. The charge density differences of the FOX-7–GO composites were primarily concentrated at the interface, especially at the hydroxyl or epoxy site. Generally, the FOX-7 accumulated more negative charges and the GO gained more positive charges, indicating that the charge transfer occurs from GO to FOX-7.

Interfacial charge transfer was characterized by Bader charge analysis to further determine the interfacial interaction strength quantitatively. As summarized in Table 1, the charges (0.036 |e|–0.059 |e|) were transferred from GO to FOX-7, where such charge transfer might enhance the interfacial interaction. In particular, the amount of charge transfer for the G–O was nearly consistent with the trend of interface binding energies, indicating that the charge transfer mainly contributed composite stability. However, the binding energy between FOX-7 and the G–OH was dominated not only by interfacial charge transfer, but also by the interfacial hydrogen bonds. For example, the comparable larger charge transfer of 0.057 |e| for the GO with the sixth type single hydroxyl group had a lower binding energy −0.58 eV.nm^−2^, whereas the GO with the first type single hydroxyl group attained the highest binding energy −0.70 eV.nm^−2^ due to its strong interfacial hydrogen bonding. For comparison, a 0.058 |e| charge transfer to FOX-7 from graphene was also observed, which coincided well with the previous computational result [19]. Consequently, the type and site of oxygen-containing groups are important for the formation of FOX-7–GO composites.

**Figure 4:**
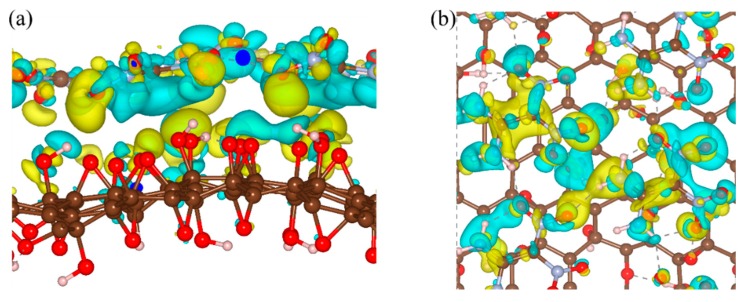
(**a**) Side view and (**b**) top view of charge density differences of the FOX-7–GO composite with OH:O = 9:5 oxygen-containing groups. Gray, red, and white balls represent carbon, oxygen, and hydrogen atoms, respectively. Isosurface levels were set at 0.0015 Bohr^−3^; cyan: charge accumulation; yellow: charge depletion.

We then investigated the interfacial interactions of FOX-7–GO composites by changing the hydroxyl and epoxy groups (OH:O) ratio at the direct contact surfaces. Seven GO models with different OH:O ratios were constructed and optimized. Intuitively, the binding energies for the seven FOX-7–GO composites were in the range of −0.76 to –1.00 eV·nm^−2^ (see Table 2), which were smaller than that of graphene–FOX-7 (−1.26 eV·nm^−2^) and G-O–FOX-7, owing to the enlarged interlayer distance. As a representative, Figure 4 shows the optimized structure and charge density differences for the FOX-7–GO composite with OH:O = 9:5. Clearly, charge redistribution mainly occurs at the interfacial region. Contrary to the GO with single hydroxyl or epoxy group, the GO interface regions of FOX-7–GO composite mainly showed charge accumulation, whereas the FOX-7 interface regions showed charge depletion. A quantitative Bader charge analysis also suggested charge transfer occurred from the FOX-7 monolayer to GO when forming the composite (see Table 2). As mentioned above, the interface binding energies were mainly determined by the number of interfacial hydrogen bonds and the charge transfer between GO and FOX-7. FOX-7–GO composites with the largest amount of transferred charge (0.276 |e|, OH:O = 0:14) or the largest number of hydrogen bonds (4, OH:O = 14:0, 5:9) will be more stable with a larger binding energy, i.e., −1.00, −0.96, and −0.98 eV·nm-^2^, respectively. Nevertheless, it should be pointed out that since the atomic structure of GO is extremely complexity and amorphous at a large length scale [31], the binding energy is also affected by the interaction sites between the amino and nitro groups of FOX-7 and the oxygen-containing groups of GO.

Assessing the performance sensitivity relationships of energetic materials is very complex and challenging [6]. By now, many experimental and theoretical models have been developed to predict the impact sensitivity of EMs, such as the drop hammer test (*h_50_*), oxygen balance, molecular properties, the ratios of C and H to O for different classes of explosive compounds, and so on [5,6,32]. Among them, oxygen balance is a practical sensitivity indicator for EMs, which can be simply derived from the stoichiometry of the energetic materials. It is noteworthy that the introduction of GO will influence the chemical composition of FOX-7, which is expected to affect the sensitivity. According to the well-established sensitivity–stoichiometry correlations defined by Storm [5], sensitivities became worse as the oxygen balance increases in organic energetic materials. Compared with that of bulk FOX-7, the oxygen balance of FOX-7–GO in this work increased in the range of 5–10%, depending on the content of oxygenic functional groups of the GO model. This certainly shed some lights on the reduction of sensitivity of FOX-7 after coating with a GO layer.

As an alternative sensitivity indicator, the band gap related to the intrinsic molecular or crystal structures is also used to measure the impact sensitivity of EMs [32]. According to this, the decrease of band gap could facilitate electrons exciting from the valence band maximum (VBM) to the conduction band minimum (CBM), thereby increasing the impact sensitivity. Here, the FOX-7 monolayer had a band gap of 2.49 eV, which was consistent with the previous standard DFT calculation results [33,34]. As can be seen in Table 1 and Table 2, in FOX-7–GO composites, the band gaps of the FOX-7 part were 2.266 ± 0.138 eV, which were slightly smaller than that of individual FOX-7 monolayer. It is known that the standard DFT within GGA always underestimates the band gaps; however, here, we only focused on the band gap variation trends of FOX-7–GO composites. Therefore, from the point of view of the band gap criterion, the impact sensitivity of the FOX-7 will be affected with the introduction of GO.

**Figure 5:**
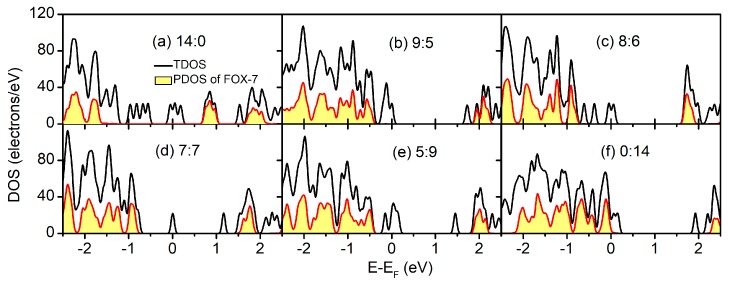
Total density of states (TDOS) and the constituent partial density of states (PDOS) of the FOX-7–GO composites with different OH:O ratios.

To elucidate the electronic structure in the FOX-7–GO composites with regard to the individual systems, the total density of states (TDOS) and the constituent partial density of states (PDOS) of the FOX-7–GO composites with different OH:O ratios are plotted in Figure 5. Because of the different electronic structures of GO with different OH:O ratios, both metallic (Figure 5a,c–f) and semiconducting (Figure 5b) behavior was found from the TDOS, and the metallic properties mainly resulted from the local defects of GO. However, for the FOX-7 part, semiconducting behavior was always found with the band gaps ranging from 2.317 eV to 2.481 eV, owing to different interfacial interactions. Basically, as shown in Table 2, the band gap of the FOX-7 part is smaller for stronger interfacial interactions, i.e., larger charge transfer and lower binding energy.

**Figure 6:**
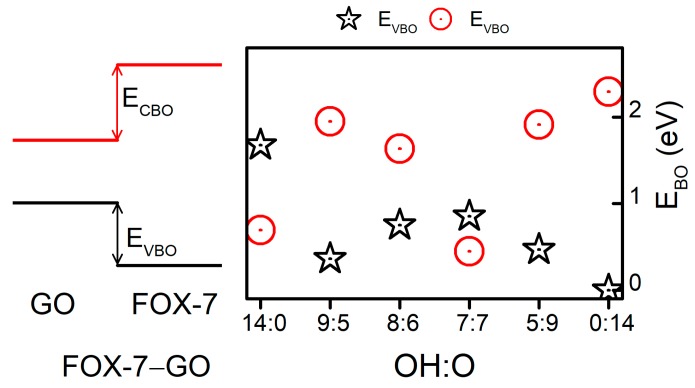
Band alignments of the FOX-7–GO interfaces. E_CBM_ is the conduction band offset and E_VBM_ is the valence band offset.

Furthermore, as shown in Figure 5, the band edges (VBM and CBM) of FOX-7 shifted with different GO interactions. In addition to the band gap variation, the valence band offset (VBO) and conduction band offset (CBO) between GO and FOX-7 may be another factor influencing the impact sensitivity. Hence, we have discussed the relative band alignments between FOX-7 and GO with different OH:O ratios. Figure 6 displays the band alignments of the FOX-7–GO interfaces. In a co-supercell, FOX-7 and GO will share the same vacuum level; the band offset can then be read directly from the PDOS [35] shown in Figure 5. Type-I band alignments between FOX-7 and GO were found and are schematically shown in the left panel of Figure 6. The band offset for the valence and conductance bands can be defined as $E_{VBO}=E_{VBM}^{GO}-E_{VBM}^{FOX-7}\;$, $E_{CBO}=E_{CBM}^{FOX-7}-E_{CBM}^{GO}\;$, respectively, in which $E_{VBM}^{FOX-7}$ and $E_{CBM}^{FOX-7}$ ($E_{VBM}^{GO}\;$, $E_{CBM}^{GO}\;$) are the VBM and CBM of FOX-7 (GO). Note that the electronic properties of GO are determined by both the OH:O ratio and their distributions, and we only considered the most thermodynamically stable configurations for the fixed OH:O ratio, which were responsible for the fluctuation in band offset as shown in Figure 6. For VBM, the band offset was found to generally decrease from 1.67 eV (OH:O = 14:0) to 0 eV (OH:O = 0:14), with the exception of OH:O = 9:5. By contrast, except for the hybrid system with OH:O = 7:7, the band offset for CBM was found to increase with increase of the OH:O ratio, i.e., from 0.69 eV (OH:O = 14:0) to 2.29 eV (OH:O = 0:14). Compared to the band gap (2.49 eV) of the FOX-7 monolayer, the smaller *E*_VBO_ (*E*_CBO_) make it easier to migrate the holes (electrons) at interlayer. As a result, we speculated that when external stimuli are injected, a GO coating layer on the surface of FOX-7 particles may reduce charge accumulation on the FOX-7 surface, thereby preventing the formation of hot spots and reducing sensitivity to some extent.

## 4. Conclusions

In summary, systematic DFT calculations were performed to investigate the relationship between interfacial interaction and electronic properties for FOX-7–GO composites. Three main conclusions were drawn as follows: (1) GO and wave-shaped FOX-7 monolayer can combine spontaneously owing to significant interfacial charge transfer and hydrogen bonds. (2) After contacting GO with different OH:O ratios, the band gaps of FOX-7 monolayer will be smaller compared with those of individual FOX-7 monolayers, because of the interlayer interactions. (3) Under external stimuli, the interfacial band alignments are beneficial for electrons (holes) migrating from GO to FOX-7, further reducing charge accumulation on the FOX-7 surface. As a result, the FOX-7–GO composites exhibit excellent thermal stability due to interfacial charge transfer and hydrogen bonds. The hydrogen bonds could break or form at interfaces and electrons transport from the surface, coating GO sheets to FOX-7, which further dissipates the mechanical stimuli energy. These results provide vital hints for interpreting experimental observations on the excellent desensitization effect of GO coating on EMs.

## Figures and Tables

**Table 1:** The interfacial hydrogen bond length (*d_HB_*), charge transfer from GO to FOX-7 monolayer (CT), binding energies (Δ*E_b_*), and band gaps (Δ*E_gap_*) of the FOX-7 monolayer in composite.

		*d_HB_*/Å	CT/e	Δ*E_b_*/eV·nm^−2^	Δ*E_gap_*/eV
FOX-7					2.490
FOX-7–G		-	−0.058	−1.26	2.394
FOX-7–G–OH	site1	1.984/1.840	−0.048	−0.70	2.224
	site2	2.097	−0.036	−0.49	2.274
	site3	-	−0.040	−0.50	2.161
	site4	2.012	−0.043	−0.61	2.128
	site5	1.979	−0.044	−0.63	2.363
	site6	-	−0.057	−0.58	2.322
FOX-7–G–O	site1	-	−0.043	−1.05	2.404
	site2	-	−0.051	−1.09	2.327
	site3	-	−0.050	−1.10	2.365
	site4	-	−0.059	−1.11	2.320
	site5	-	−0.055	−1.11	2.339
	site6	-	−0.050	−1.07	2.295
	site7	-	−0.048	−1.03	2.317
	site8	-	−0.047	−1.04	2.322
	site9	-	−0.041	−1.06	2.326
	Site10	-	−0.054	−1.10	2.290

**Table 2:** The number of interfacial hydrogen bonds (*N_HB_*), charge transfer from GO to FOX-7 monolayer (CT), binding energies (Δ*E_b_*), and band gaps (ΔE_gap_) of FOX-7 monolayer in composites with different OH:O ratios.

	OH:O	*N_HB_*	CT/e	Δ*E_b_*/eV⋅nm^−2^	ΔE_gap_ /eV
FOX-7–G–OH	14:0	4	0.061	−0.96	2.444
	9:5	2	0.045	−0.75	2.403
	8:6	2	0.038	−0.76	2.481
	7:7	2	0.042	−0.86	2.459
	5:9	4	0.067	−0.98	2.387
	1:13	1	0.062	−0.83	2.317
	0:14	0	0.276	−1.00	2.371
